# The benefits of a department-wide prehabilitation program: A propensity score match analysis

**DOI:** 10.1016/j.surg.2025.109489

**Published:** 2025-06-12

**Authors:** Shaleen V. Sathe, Yijin Huang, Jorge G. Zarate Rodriguez, Heidy Cos, Jennifer Cook, Melanie Koenen, William C. Chapman, Benjamin D. Kozower, Ryan C. Fields, Dominic E. Sanford

**Affiliations:** a Department of Surgery, Barnes-Jewish Hospital and Washington University School of Medicine, Saint Louis, MO; b Department of Surgery, Wake Forest University School of Medicine, Winston-Salem, NC; c Alvin J. Siteman Cancer Center, Washington University School of Medicine, Saint Louis, MO

## Abstract

**Background:**

Despite increasing use of prehabilitation in aging surgical patients, large studies demonstrating benefits on postoperative outcomes are lacking. This study aimed to determine if a department-wide prehabilitation program (Surgical Prehabilitation and Readiness) improves 30-day mortality, discharge to post-acute care facilities, and postoperative length of stay in high-risk surgical patients compared to historical controls, and to examine age’s influence on these outcomes.

**Methods:**

Surgical Prehabilitation and Readiness patients with 30 days of postoperative follow-up were compared to patients from the National Surgical Quality Improvement Program database undergoing procedures at the same institution during the 5 years before Surgical Prehabilitation and Readiness implementation (pre–Surgical Prehabilitation and Readiness). Surgical Prehabilitation and Readiness patients were propensity score matched to pre–Surgical Prehabilitation and Readiness patients in a 1:3 ratio, and outcomes were compared.

**Results:**

Over 40 months, 424 patients completed Surgical Prehabilitation and Readiness and underwent surgery with 30 days of follow-up. Compared with pre–Surgical Prehabilitation and Readiness patients, Surgical Prehabilitation and Readiness patients were significantly older (median: 69.9 vs 60.7 years, *P* < .001) with higher American Society of Anesthesiologists class (≥3: 84.7% vs 54.4%, *P* < .001) and more comorbidities. Compared to propensity score–matched pre–Surgical Prehabilitation and Readiness patients (*n* = 1,161), Surgical Prehabilitation and Readiness patients (*n* = 387) had significantly decreased 30-day mortality (0.8% vs 2.8%, *P* = .023), discharge to post-acute care facility (8.8% vs 12.9%, *P* = .030), and length of stay (7.2 vs 8.0 days, *P* = .039). Older Surgical Prehabilitation and Readiness patients (age > median) exhibited significantly decreased 30-day mortality (0.6% vs 3.3%, *P* = .044) and discharge to post-acute care facility (11.6% vs 19.3%, *P* = .017), whereas younger Surgical Prehabilitation and Readiness patients (age ≤ median) exhibited decreased length of stay (6.9 vs 8.2 days, *P* = .021).

**Conclusions:**

Prehabilitation reduces postoperative mortality, loss of functional independence, and hospital recovery time, and the benefits vary by age. These findings support the implementation of prehabilitation programs in clinical practice.

## Introduction

The risk of postoperative morbidity and mortality increases significantly with advancing age and declining functional status.^1–3^ In the United States, the population aged 65 years and older is expected to double in the next 40 years, and a substantial portion of surgical procedures will be performed on individuals in this age group.^4–6^ Additionally, more than 70% of Americans aged 65 years and older have at least 1 chronic comorbid condition, with 50% having multiple,^7^ and the burden of comorbidities is predicted to continue to rise.^8^ As surgical patients become older, frailer, and higher-risk,^9^ ongoing efforts to identify interventions that optimize their functional status are essential.

The preoperative period may be an effective time and opportunity for intervention to enhance patients’ functional capacity. Prehabilitation uses this strategy of targeting the preoperative period to optimize patients for surgery. There continues to be discussion regarding the most effective methods for implementing a successful prehabilitation program and identifying the patients who would benefit the most. Evidence suggests that prehabilitation can improve preoperative functional status, decrease postoperative complications,^10–17^ and improve surgical outcomes.^16,18–21^ However, current recommendations for the integration of prehabilitation into clinical practice remain limited because of heterogenous study designs and insufficient study power.^22–25^ Additionally, large-scale studies that show improvement of post-operative mortality are lacking.^24,26^ Thus, establishing clear evidence to incentivize payers and other stakeholders to support prehabilitation programs is needed.

This study evaluates the impact of a department-wide multimodal prehabilitation program called Surgical Prehabilitation and Readiness (SPAR). Patients enrolled in SPAR were compared to historical control patients from the institutional American College of Surgeons National Surgical Quality Improvement Program (ACS NSQIP) database (pre-SPAR). Propensity score matching was used to compare postoperative outcomes between the 2 study groups. The purpose of the study was to determine whether SPAR decreases 30-day mortality, need for discharge to post-acute care facilities (PACFs), and postoperative length of stay (LOS) in high-risk surgical patients compared with historical control patients and to determine the difference in outcomes based on age group. The future of surgical practice and the health care system will greatly benefit from understanding the impact of prehabilitation on patient outcomes.

## Methods

### The Prehabilitation Program

The SPAR program is a department-wide multimodal prehabilitation initiative targeting 4 domains with evidence-based interventions^27–34^: physical activity/function, pulmonary health, nutrition, and mindfulness/mindset.^35^ The program was developed as a quality improvement initiative. Although the program targets patients older than 70 years, any patient determined to be high-risk by their surgeon is eligible for enrollment in SPAR. Additional criteria for patient referral are planned inpatient surgery with an expected postoperative LOS of 2 days or more and a minimum preoperative enrollment time of 14 days. Patients are not automatically enrolled but rather are selectively referred based on surgeon discretion. Most high-risk patients are referred to SPAR by their individual surgeon after an outpatient clinic visit.

Details about the individual components of the SPAR program have been previously described by our group^35^ and are summarized in Supplementary Figure S1. Briefly, to promote physical activity, all patients receive wearable accelerometers connected to their smartphones and are encouraged to walk at least 5,000 steps daily, which is supported by automated text message reminders. Patients are referred to physical therapy for evaluation. For respiratory fitness, patients are given an incentive spirometer with instructions for use, and patients who smoke are offered smoking cessation counseling and pharmacotherapy as needed. To optimize nutrition, patients are educated on healthy eating habits, and those who screen positive for malnutrition are referred to a registered dietitian. SPAR patients are assessed for anxiety and depression using validated questionnaires, encouraged to begin gratitude journaling, and counseled on maintaining a positive mindset before surgery. A SPAR team member, nurse or medical assistant, monitors progress through weekly phone calls. The program concludes on the date of surgery or if surgery is no longer being considered.

### Data Sources and Collection

Over a period of 40 months, patient demographics, comorbidities, surgical details, and 30-day postoperative outcomes were tracked and prospectively recorded in a secure RedCap database. SPAR patients were compared to all patients in the ACS NSQIP database who underwent elective procedures of the same *Current Procedural Terminology* (*CPT*) code at the same institution during the 5 years (2016–2020) prior to the implementation of the SPAR program. A historical control group was used to minimize selection bias. The complete list of *CPT* codes used, compiled based on previously published literature using the ACS NSQIP database, is available in Supplementary Table S1. Patients who underwent procedures that are not included in the NSQIP database (eg, liver transplantation) were excluded from the analysis. This study was approved by the Institutional Review Board (IRB).

### Propensity Score Matching and Analysis

Patients enrolled in SPAR who underwent surgery and had at least 30 days of postoperative follow-up at the time of the analysis were propensity score matched to pre-SPAR NSQIP patients in a 1:3 ratio. The following variables were used for matching and were chosen because of their inclusion in the ACS NSQIP Risk Calculator^36^: age, sex, non-White race, smoking status, functional status, American Society of Anesthesiologists (ASA) class, dyspnea, obesity, diabetes, steroid use, hypertension, congestive heart failure, chronic obstructive pulmonary disease, disseminated cancer, and surgery type. Propensity score matching was performed using a multivariable logistic regression model. A greedy matching algorithm with a nearest neighbor approach and a caliper distance of 0.2 standard deviations of the logit of the propensity score^37^ were used. The balance between matched pairs was assessed using standardized differences, with a standardized difference of less than 10% indicating excellent balance.^38^ An assessment of the predictive ability of the propensity score model is provided as Supplementary Figure S2. Missing data in propensity score estimation were excluded from the final analysis.

A subgroup analysis was performed to compare the outcomes of older and younger SPAR patients. The study cohort was divided into 2 groups: older patients with ages greater than the median age of 69.9 years, and younger patients with ages less than or equal to the median age. This cutoff age of 69.9 years was also consistent with the commonly accepted age of 70 years at which age-related organ dysfunction and comorbidities tend to develop.^39^ These 2 groups were independently propensity score matched using the same described method, and similar subsequent statistical analysis was completed.

An unpaired Student’s *t* test was used to compare independent normally distributed continuous variables and outcomes, whereas the Wilcoxon rank-sum test was used for non-normally distributed continuous variables and outcomes. The χ^2^ test was used to compare categorical variables and outcomes. All *P* values were 2-sided.

### Outcomes

The primary endpoint of the study was 30-day mortality, whereas secondary endpoints included discharge to a post-acute care facility (ie, skilled nursing facility or inpatient rehabilitation facility), 30-day readmission, and postoperative LOS. NSQIP definitions were used for all comorbidities and outcomes.^36^

## Results

### Study cohort

**Figure 1.**
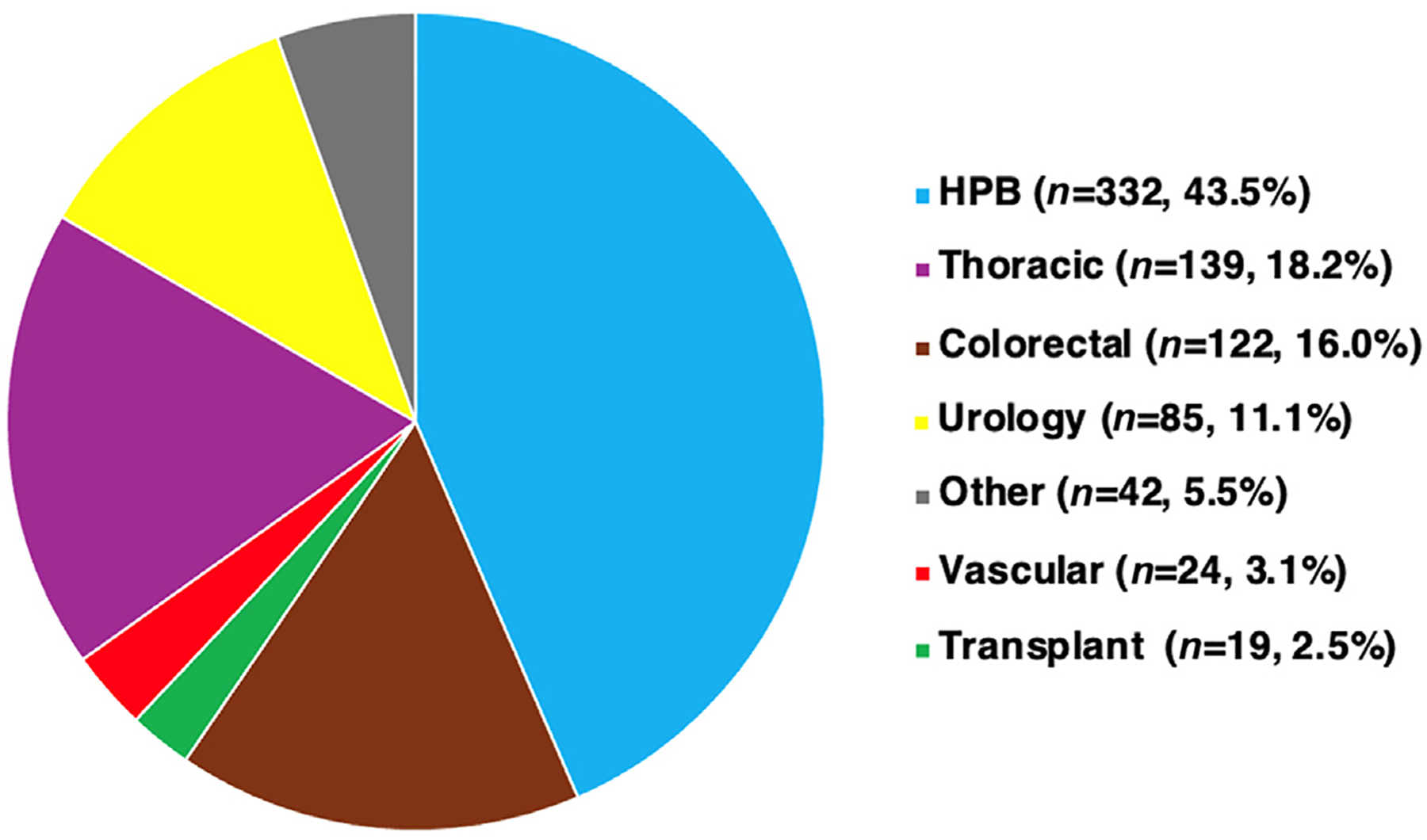
Surgical Prehabilitation and Readiness (SPAR) patient enrollment by surgical division. *HPB*, hepatopancreatobiliary.

Over the 40-month study period, 763 patients were enrolled in SPAR. The most common service lines to refer patients to the program were Hepatobiliary and Pancreatic Surgery (*n* = 332, 43.6%), Thoracic Surgery (*n* = 139, 18.2%) Colorectal Surgery (*n* = 122, 16.0%), Urology (*n* = 85, 11.1%), and Vascular Surgery (*n* = 24, 3.1%) (Figure 1). One hundred ninety patients did not undergo surgery and were also excluded from the final analysis. The reasons for patients not undergoing surgery were as follows: change in surgical plan (*n* = 65), progression of disease (*n* = 51), the patient opted to not have surgery (*n* = 46), deceased patient (*n* = 16), and other miscellaneous reasons (*n* = 12). Fifty-four patients were lost to follow-up and un-enrolled from SPAR. Thirty-one patients were waiting for surgery at the time for analysis; 20 of these patients were undergoing neoadjuvant chemotherapy.

At the time of analysis, a total of 424 patients had undergone surgery and had at least 30 days of postoperative follow-up. The most common categories of operations performed were pancreaticoduodenectomy (*n* = 67), cystectomy (*n* = 55), minimally invasive colectomy (*n* = 44), distal pancreatectomy and splenectomy (*n* = 37), and esophagectomy (*n* = 32). There was a total of 42 different types of operations performed in the study group. The median age of these SPAR patients was 69.9 years, 188 (44.3%) were female, and 259 (84.7%) had a severe systemic disease (ASA class ≥3) (Table I). One hundred forty-six patients (34.4%) had undergone neoadjuvant chemotherapy. The median time from SPAR enrollment until surgery was 44 (interquartile range 30–74) days.

### Comparison to historical control patients in the NSQIP database

Compared to historical control patients within the institution’s 2016–2020 NSQIP database who underwent the same operations before SPAR implementation (pre-SPAR patients, *n* = 5,989), SPAR patients were significantly older (68.5 vs 58.7, *P* < .001) with a higher ASA class (ASA class ≥3: 84.7% vs 54.4%, *P* < .001). Additionally, SPAR patients were significantly more likely to have a history of dyspnea (31.6% vs 12.5%, *P* < .001), diabetes (23.1% vs 16.4%, *P* < .001), steroid use (15.6% vs 7.2%, *P* < .001), hypertension (62.0% vs 50.3%, *P* < .001), congestive heart failure (10.6% vs 1.4%, *P* < .001), and chronic obstructive pulmonary disease (13.7% vs 6.3%, *P* < .001) (Table I). SPAR patients were also less likely to be functionally independent compared with control patients (independent function: 96.7% vs 98.5%, *P* = .005). Compared with the pre-SPAR cohort, SPAR patients were more likely to undergo pancreaticoduodenectomy, distal pancreatectomy and splenectomy, cystectomy, esophagectomy, total and distal gastrectomy, small bowel resection, resection of retroperitoneal mass, mediastinal tumor resection, infected graft excision, scrotal repair, ureteral repair, hyperthermic intraperitoneal chemotherapy, heart valve replacement, and wound debridement. They were less likely to undergo incisional hernia repair with mesh, open abdominal aortic aneurysm repair, major hepatectomy, thyroidectomy, laparoscopic prostatectomy, laparoscopic appendectomy, and mastectomy (Table I). Although SPAR patients had significantly increased LOS (mean 6.9 vs 5.2 days, *P* < .001) compared with pre-SPAR patients, there were no significant differences in 30-day mortality (0.7% vs 1.1%, *P* =.416), need for discharge to PACF (8.5% vs 9.5%, *P* = .512), or 30-day readmission (14.4% vs 11.4%, *P* = .067) (Supplementary Figure S3).

### Propensity score matching: SPAR versus Pre-SPAR patients

**Figure 2.**
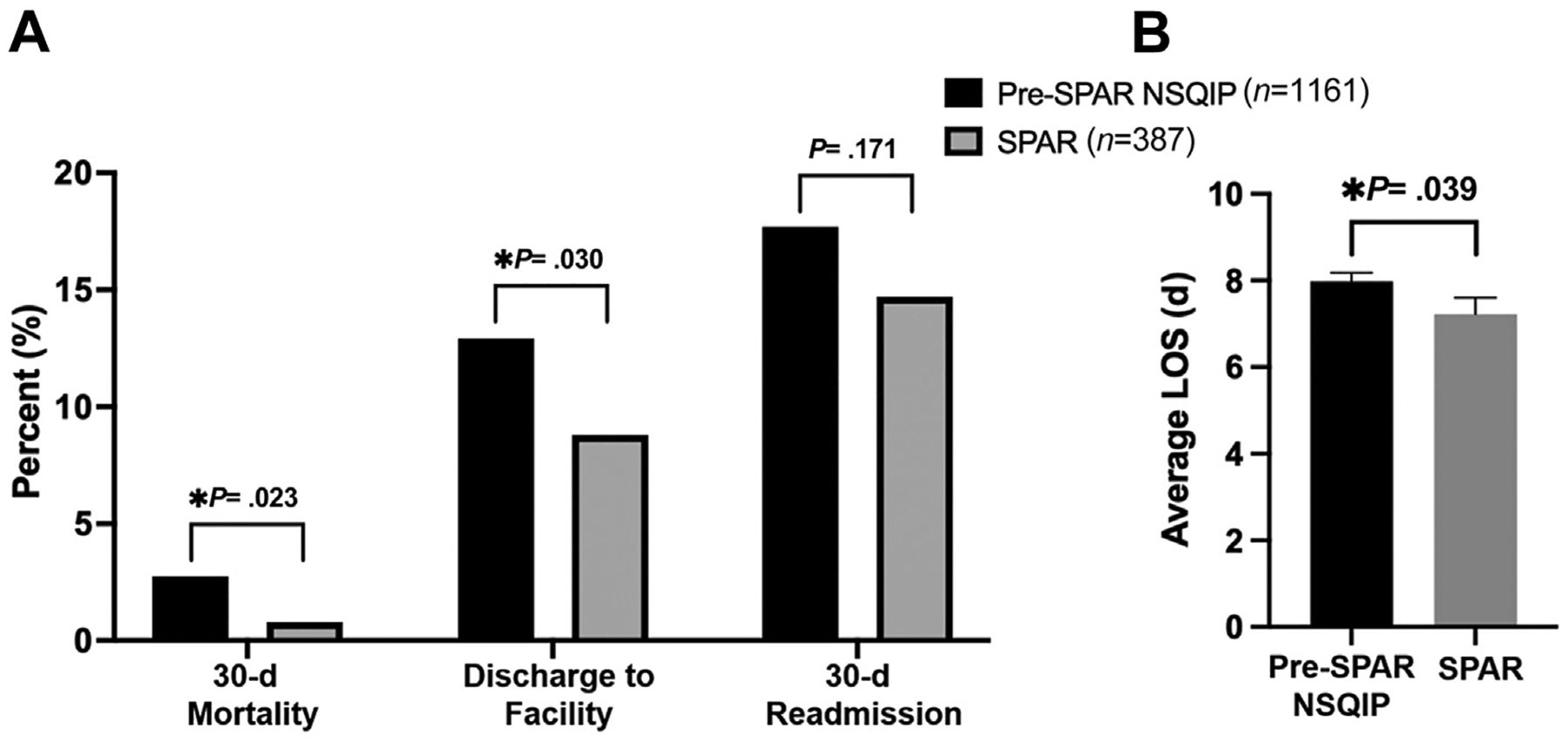
Patient outcomes of Surgical Prehabilitation and Readiness (SPAR) patients (*n* = 387) compared with propensity score–matched historical controls (*n* = 1,161). (A) 30-day mortality, discharge to facility, and 30-day readmission. (B) Average postoperative length of stay (LOS) with standard errors bars.

SPAR patients were propensity score matched to the pre-SPAR patients in a 1:3 ratio. Three hundred eighty-seven patients (91.3%) were successfully matched to 1,161 pre-SPAR patients with standardized differences of less than 10% for all matched variables, indicating excellent balance between the groups (Table II). In this matched analysis, SPAR patients had significantly decreased 30-day mortality (0.8% vs 2.8%, *P* = .023), decreased need for discharge to PACF (8.8% vs 12.9%, *P* = .030), and decreased postoperative LOS (mean 7.2 vs 8.0 days, *P* = .039) compared with historical control patients. There was no significant difference in 30-day readmission rates (14.7% vs 17.7%, *P* = .171) between the 2 groups (Figure 2). The causes of 30-day mortality in the SPAR group were septic shock (*n* = 1), postoperative hemorrhage (*n* = 1), and not documented/unknown (*n* = 1). The causes of 30-day mortality in the pre-SPAR group were cardiopulmonary complication (*n* = 12), post-operative hemorrhage (*n* = 6), septic shock (*n* = 4), cerebrovascular accident (*n* = 1), failure to thrive or advanced metastatic cancer (*n* = 1), and not documented/unknown (*n* = 8).

### Subgroup analysis by age

There was a wide range in the ages of SPAR patients, from 34 to 91 years with a median age of 69.9 years. We divided the patients into 2 groups (ie, older and younger) based on the median age of the SPAR cohort. Both groups of patients were independently propensity score matched to pre-SPAR NSQIP patients in a 1:3 ratio with less than 10% standard differences for all matched variables (Supplementary Tables S2 and S3) The older group contained patients with ages greater than the median of 69.9 years, with a total of 181 patients. The younger group contained patients with less than or equal to the median age of 69.9 years, with a total of 198 patients.

**Figure 3.**
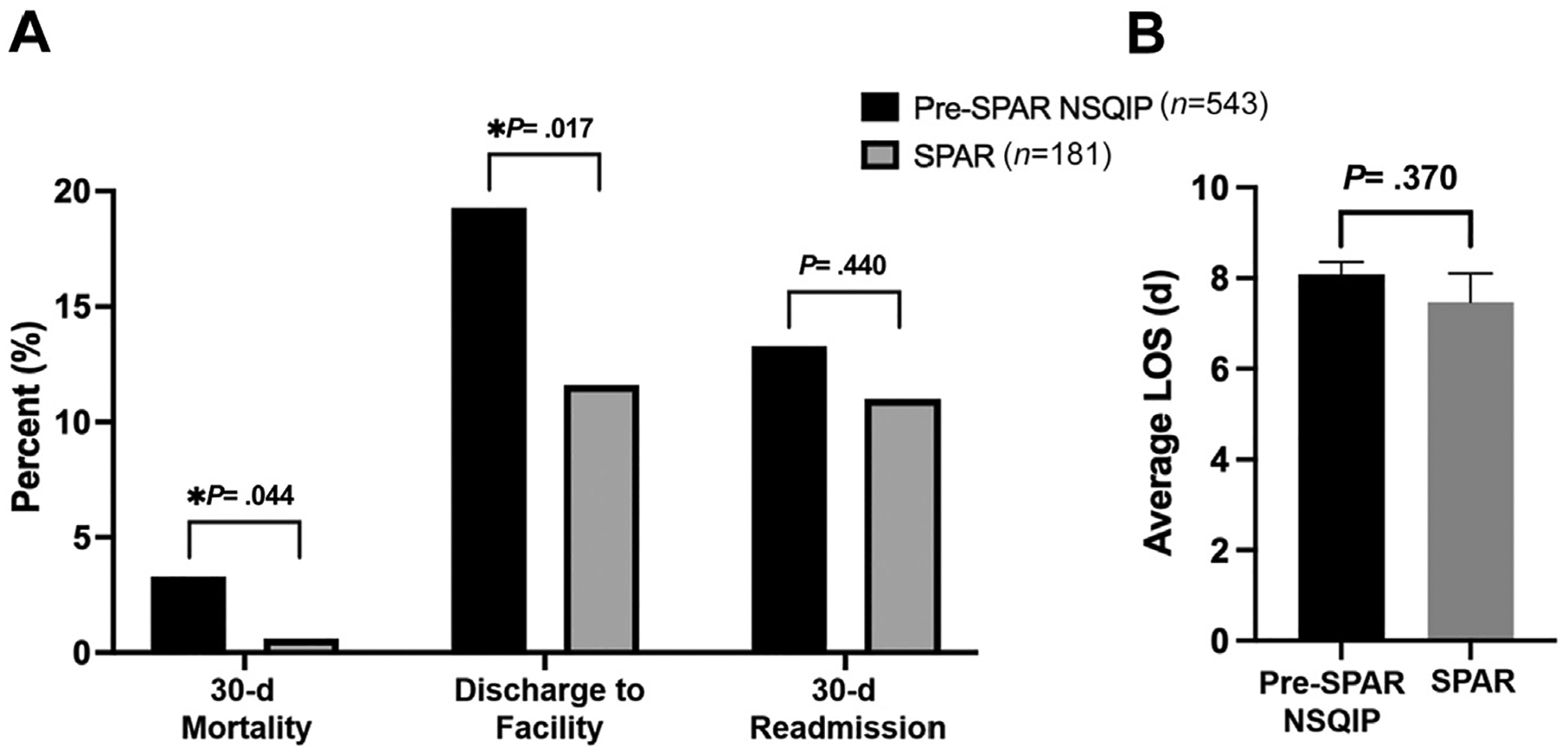
Outcomes of Surgical Prehabilitation and Readiness (SPAR) patients (*n* = 181) greater than the median age (69.9 years) compared with propensity score–matched historical controls (*n* = 543). (A) 30-day mortality, discharge to facility, and 30-day readmission. (B) Average postoperative length of stay (LOS) with standard errors bars.

**Figure 4.**
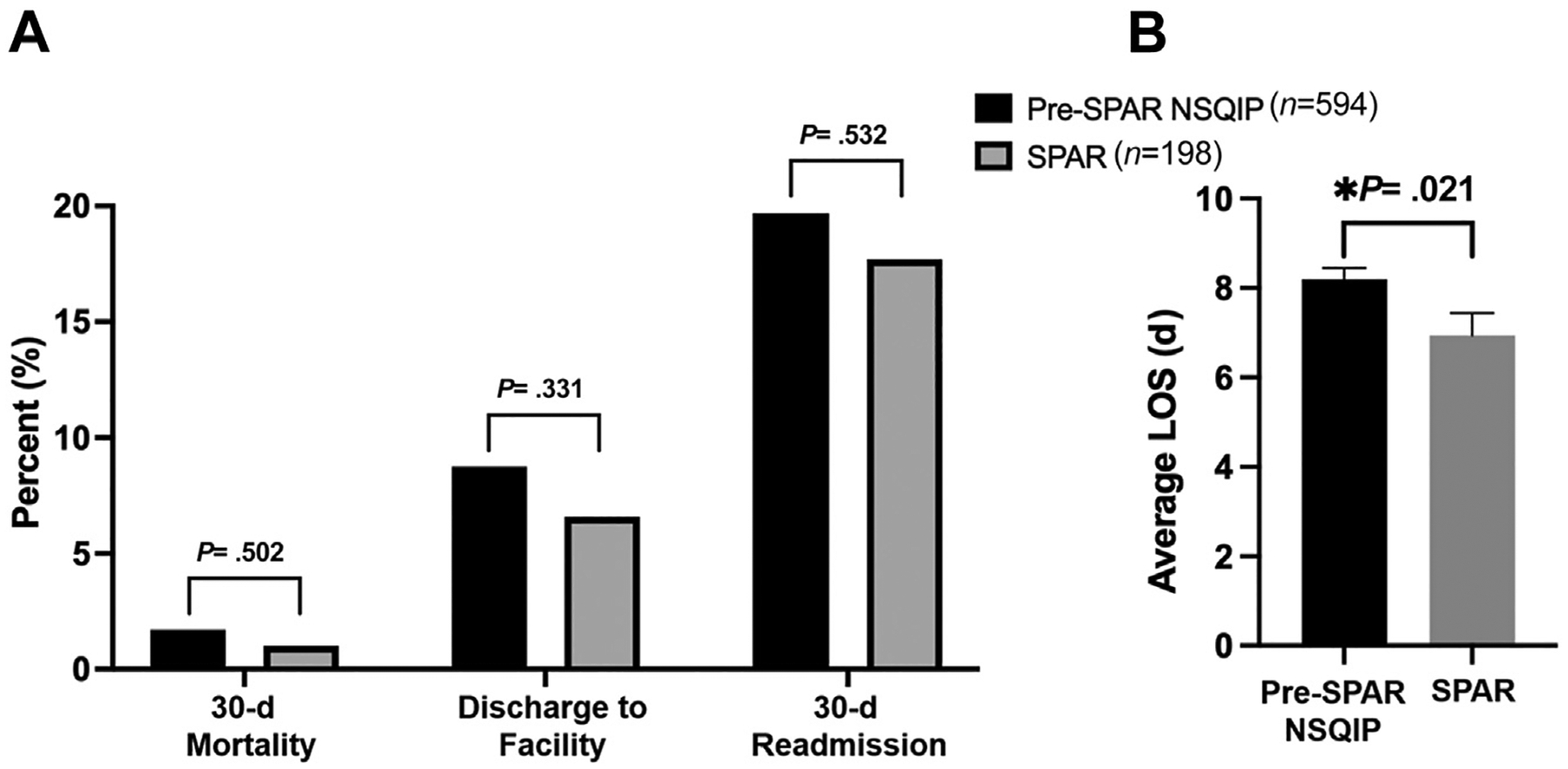
Outcomes of Surgical Prehabilitation and Readiness (SPAR) patients (*n* = 198) less than or equal to the median age (69.9 years) compared with propensity score–matched historical controls (*n* = 594). (A) 30-day mortality, discharge to facility, and 30-day readmission. (B) Average postoperative length of stay (LOS) with standard errors bars.

SPAR patients in the older age group had significantly decreased 30-day mortality (0.6% vs 3.3%, *P* = .044) and need for discharge to PACF (11.6% vs 19.3%, *P* = .017) compared with matched pre-SPAR patients. There was no significant difference in 30-day readmission (11.0% vs 13.3%, *P* = .440) or LOS (mean 7.5 vs 8.1, *P* = .370) between the 2 groups (Figure 3). SPAR patients in the younger age group had significantly decreased LOS (mean 6.9 days vs 8.2, *P* =.021) compared with matched pre-SPAR patients. There were no significant differences in need for discharge to PACF (6.6% vs 8.8%, *P* = .331), 30-day readmission (17.7% vs 19.7%, *P* = .532), or 30-day mortality (1.0% vs 1.7%, *P* = .502) between the 2 groups (Figure 4).

## Discussion

As one of the largest prospective studies evaluating a prehabilitation program to date, our results show that SPAR decreases 30-day mortality, need for discharge to PACF, and postoperative LOS in high-risk surgical patients. In older patients, SPAR reduces mortality and increases rates of discharge home. In younger patients, SPAR reduces recovery time in the hospital. Additionally, to the authors’ knowledge, this study was one of the largest to show an improvement in mortality; previous trials and prospective studies have either shown no difference in mortality^15,16,26^ or not examined mortality as an outcome.^14,18,21,40–43^ Furthermore, unlike prior studies that focused on a single type of operation, such as colorectal surgery,^11,12,16,19,20,26,41,44^ our cohort included patients undergoing a broad range of high-risk procedures, including pancreaticoduodenectomy, radical cystectomy, and esophagectomy. This higher-acuity population may explain the observed mortality benefit not previously reported in prehabilitation literature. Our findings highlight the importance of integrating prehabilitation into standard clinical practice, offering evidence for its role in optimizing both younger and older patients to improve post-operative outcomes.

We previously demonstrated that SPAR is cost-effective and feasible with reported high adherence rates.^35^ The fixed materials cost per patient was calculated to be about $40 and was found to be financially feasible during an initial pilot of SPAR. A 6-month compliance audit demonstrated an overall adherence rate of 89%, which may play a critical part in the program’s success. In this previous pilot study, the sample size was insufficient to detect differences in certain postoperative outcomes, such as hospital LOS. However, with the larger cohort in the current study, a more comprehensive analysis of outcomes was possible. Additionally, the increased power of this study allowed a clearer assessment of how prehabilitation may benefit patients based on age group.

Much of the existing literature evaluating the effects of prehabilitation has not differentiated patients based on age or functional status, making interpretation of the results difficult.^12,26,43^ The benefit of prehabilitation may depend on patient selection, as older patients with comorbid conditions might benefit differentially from a prehabilitation program compared with younger, healthier patients with less room for functional improvement. Most previous studies have targeted elderly, frail patients, who are known to be at higher risk for postoperative complications,^14,21,41,42^ including functional decline and mortality. The SPAR program was also initially designed for patients aged 70 years and older. However, in response to departmental interest in enrolling younger but high-risk patients who might also benefit from prehabilitation, the eligibility criteria were expanded. This shift in enrollment played a role in our decision to evaluate the program’s clinical impact in a broader surgical population and to differentiate by age group, particularly to inform resource allocation for patients outside the original target demographic. To the authors’ knowledge, this is the first prospective study evaluating how the impact of prehabilitation may differ based on the age group of the patient. Our study finds that SPAR reduces mortality and preserves functional independence (eg, allows discharge home) in older patients. With younger patients, who are generally at lower risk of mortality and functional decline, SPAR aids in faster recovery (eg, reduces hospital LOS). The patient populations evaluated in previous studies have been heterogeneous, and the primary outcomes examined may not have been applicable to all age groups. This heterogeneity could be a reason why prehabilitation has previously been shown to have mixed results. Improvements in patient selection and enrollment in programs like SPAR may lead to optimization of postoperative outcomes seen from prehabilitation.

Our results are consistent with those reported by Mouch et al, who found that implementation of a similarly structured multimodal prehabilitation program led to reduced postoperative LOS, higher rates of discharge home, and decreased treatment costs.^40^ Also similar to our findings with SPAR, however, this group found no improvement in readmission rates in patients undergoing prehabilitation when compared to matched control patients,^40^ and several other studies have had comparable results.^41,44,45^ In both studies, operation-specific complication rates were not evaluated. Recent retrospective data have shown that an average preoperative daily step count greater than 7,500 steps decreases the odds of adverse outcomes postoperatively,^46^ but further analysis on specific surgical outcomes was not performed. In another trial, prehabilitation was demonstrated to lower the rate of medical (eg, cardiopulmonary) complications only and no difference in number of surgical complications.^16^ In our study, 3 patients in the SPAR group died within 30 days of surgery, compared with 32 in the pre-SPAR NSQIP group, with cardiopulmonary complications being the most common cause in the latter. Notably, no cardiopulmonary deaths occurred in the SPAR group, suggesting that prehabilitation may help reduce such complications, although the current study lacks statistical power to confirm this conclusion. Future investigation with a larger sample size is needed to better understand the impact of prehabilitation on specific postoperative complications.

Previous studies on prehabilitation programs that found no improvement in outcomes did not compare prehabilitation to the existing standard of care.^41,42^ For example, McIsaac et al^42^ compared patients in a home-based total body exercise prehabilitation program to patients who were given written exercise and nutrition guidelines. The group found no differences in the six-minute walk test, hospital LOS, discharge to facility, readmissions, total hospital costs, or complications. Similarly, Carli et al^41^ compared a prehabilitation program to a postoperative rehabilitation program and found no differences in postoperative complication rate, LOS, readmission, postoperative walking capacity, or patient-reported outcome measures. However, because prehabilitation, rehabilitation, and written educational guidelines are all normally not included in standard care for most surgical patients, the generalizability of these results may be limited. A notable strength of our study is the comparison of patients undergoing prehabilitation with a historical control group from the same medical system, who had received standardized preoperative and postoperative care through the years. This approach revealed substantial improvements across a comprehensive set of postoperative outcomes.

The current study has several limitations. First, the 2 patient populations compared (pre-SPAR NSQIP patients and SPAR patients) underwent surgery at different times (2016–2020 and 2021 to present, respectively), potentially introducing bias from timing and variations in surgeon and clinical experience. However, the study was purposefully designed in this way to reduce selection bias as we aimed to identify high-risk patients similar to the SPAR cohort that did not have the option of the SPAR program. Although the 2 groups underwent a rigorous matching process to control for differences in covariates, there still remains the possibility for residual confounding from variables not included in the matching process (eg, neoadjuvant chemotherapy and radiation) and from changes in standard of care between the 2 time periods. A randomized trial design could limit this type of bias but would be difficult to design given the heterogeneity of our patient cohort and wide range of procedure types. There was a subset of patients who were enrolled in SPAR but did not undergo surgery and were not included in the final analysis. This could also be another source of selection bias as these patients were not included in the current study. Further investigation is needed to better understand why this group of patients ultimately do not undergo surgery, and what interventions, if any, could help these patients make it to surgery. Although the SPAR program targeted older and higher-risk patients, the decision for referral was ultimately up to the individual surgeons, which could also bias study results. Although a 6-month compliance audit was performed, continued assessment of adherence could not be completed as the program grew due to resource limitations. A subset of our patients was managed on enhanced recovery pathways postoperatively (eg, pancreatectomy and colorectal patients), and any potential improvement in post-operative outcomes from this program independent of SPAR was not controlled for, as data in the pre-SPAR NSQIP database were incompletely tracked. However, patients in our control group from the years prior to SPAR were also managed on similar enhanced recovery postoperative pathways. Lastly, because of a lack of power, we could not evaluate individual complications specific to the type of operation. The impact of SPAR on individual surgical complications is an area of interest for future investigation.

In conclusion, in a much larger cohort than previously studied, the current study demonstrates that SPAR, a department-wide multimodal prehabilitation program, significantly reduces 30-day postoperative mortality, need for discharge to post-acute care facilities, and postoperative LOS. Furthermore, the benefit of SPAR on postoperative outcomes differs by age, with older patients exhibiting reduced mortality and preserved functional independence and younger patients spending less time recovering in the hospital after surgery. These findings support the implementation of prehabilitation programs in clinical practice.

## Supplementary Material

1

Supplementary material associated with this article can be found, in the online version, at [https://doi.org/10.1016/j.surg.2025.109489].

## Figures and Tables

**Table I:** Characteristics of SPAR patients and Pre-SPAR NSQIP patients

Characteristic	SPAR patients (*n* = 424)	Pre-SPAR NSQIP patients (*n* = 5,989)	*P* value
Age, y, median (IQR)	69.9 (62.9–75.9)	60.7 (50.0–69.2)	<.001
Sex: female, *n* (%)	188 (44.3)	2,881 (48.1)	.147
Non-White race, *n* (%)	49 (11.6)	873 (14.6)	.066
Smoking, *n* (%)	98 (23.1)	1,198 (20.0)	.139
ASA status ≥3, *n* (%)	359 (84.7)	3,259 (54.4)	<.001
Independent function, *n* (%)	410 (96.7)	5,899 (98.5)	.005
Obesity, *n* (%)	167 (39.4)	2,355 (39.3)	>.999
Dyspnea, *n* (%)	134 (31.6)	750 (12.5)	<.001
Diabetes, *n* (%)	98 (23.1)	984 (16.4)	<.001
Steroid use, *n* (%)	66 (15.6)	430 (7.2)	<.001
Hypertension, *n* (%)	263 (62.0)	3,012 (50.3)	<.001
Congestive heart failure, *n* (%)	45 (10.6)	83	<.001
COPD, *n* (%)	58 (13.7)	375 (6.3)	<.001
Disseminated cancer, *n* (%)	22 (5.2)	475 (7.9)	.052
Operation type, n (%)			
Pancreaticoduodenectomy	67 (15.8)	440 (7.3)	<.001
Distal pancreatectomy and splenectomy	37 (8.7)	203 (3.4)	<.001
MIS colectomy	44 (10.4)	674 (11.3)	.636
Open colectomy	15 (3.5)	230 (3.8)	.855
Open rectal resection	6	162	.147
MIS rectal resection	10	93	.282
Incisional hernia repair with mesh	12	732 (12.2)	<.001
Cystectomy	55 (13.0)	220 (3.7)	<.001
Esophagectomy	32 (7.5)	229 (3.8)	<.001
Open abdominal aortic aneurysm repair	4	159	.045
Deep inferior epigastric perforator flap	3	71	.512
Total gastrectomy	7	7	<.001
Distal gastrectomy	14 (3.3)	22	<.001
Major hepatectomy	3	147	.006
Partial hepatectomy	17 (4.0)	249 (4.2)	.845
Ostomy takedown	6	72	.875
Small bowel resection	11	58	.004
Resection of retroperitoneal mass	13 (3.1)	22	<.001
Lung resection	9	202 (3.4)	.210
Mediastinal tumor resection	1	0	<.001
Laparoscopic hiatal hernia repair	16	131	.052
Aortobifemoral bypass	1	64	.161
Bilateral salpingo-oophorectomy	1	36	.530
Endovascular aortic procedure	6	102	.803
Hepaticojejunostomy	2	11	.474
MIS adrenalectomy	1	25	.863
Laparoscopic cholecystectomy	10	202 (3.4)	.323
Thyroidectomy	1	391 (6.5)	<.001
Excision of infected graft	1	0	<.001
Open inguinal hernia repair	3	102	.173
Laparoscopic prostatectomy	1	359 (6.0)	<.001
Lysis of adhesions	2	19	.922
Superficial lymphadenectomy	2	7	.224
Scrotal repair	1	0	<.001
Laparoscopic appendectomy	2	275 (4.6)	<.001
Open appendectomy	1	14	>.999
Ureteral repair	1	0	<.001
Panniculectomy	1	91	.053
Mastectomy	1	168	.002
Hyperthermic intraperitoneal chemotherapy	1	0	<.001
Heart valve replacement	2	0	<.001
Wound debridement	1	0	<.001

*ASA*, American Society of Anesthesiologists; *COPD*, chronic obstructive pulmonary disease; *IQR*, interquartile range; *MIS*, minimally invasive surgery; *SD*, standard deviation; *SPAR*, Surgical Prehabilitation and Readiness.

**Table II:** Characteristics of SPAR patients and propensity score–matched Pre-SPAR NSQIP patients

Characteristic	SPAR patients (*n* = 387)	Matched pre-SPAR NSQIP patients (*n* = 1,161)	Standardized difference, %
Age, y, mean (SD)	68.1 (11.1)	67.4 (11.7)	6.1
Sex: female, *n* (%)	167 (43.2)	500 (43.1)	0.2
Non-White race, *n* (%)	41 (10.6)	131 (11.3)	2.2
Smoking, *n* (%)	89 (23.0)	261 (22.5)	1.2
ASA status ≥3, *n* (%)	325 (84.0)	936 (80.6)	8.8
Independent function, *n* (%)	375 (96.9)	1,130 (97.3)	2.6
Obesity, *n* (%)	154 (39.8)	452 (38.9)	1.8
Dyspnea, *n* (%)	114 (29.5)	321 (27.6)	4.0
Diabetes, *n* (%)	92 (23.8)	269 (23.2)	1.4
Steroid use, *n* (%)	54 (14.0)	142 (12.2)	5.1
Hypertension, *n* (%)	235 (60.7)	715 (61.6)	1.8
Congestive heart failure, *n* (%)	35 (9.0)	76 (6.5)	9.3
COPD, *n* (%)	49 (12.7)	143 (12.3)	1.0
Disseminated cancer, *n* (%)	20 (5.2)	64 (5.5)	1.5
Operation type, *n* (%)			
Pancreaticoduodenectomy	67 (17.3)	187 (16.1)	3.2
Distal pancreatectomy and splenectomy	35 (9.0)	96 (8.3)	2.8
MIS colectomy	42 (10.9)	138 (11.9)	3.3
Open colectomy	14 (3.6)	42 (3.6)	0.0
Open rectal resection	6	21	2.0
MIS rectal resection	10	25	2.8
Incisional hernia repair with mesh	12 (3.1)	47 (4.0)	5.1
Cystectomy	53 (13.7)	145 (12.5)	3.6
Esophagectomy	32 (8.3)	98 (8.4)	0.6
Open abdominal aortic aneurysm repair	4	11	0.9
Deep inferior epigastric perforator flap	3	16	5.8
Total gastrectomy	5	6	8.2
Distal gastrectomy	9	19	4.9
Major hepatectomy	3	14	4.3
Partial hepatectomy	17 (4.4)	51 (4.4)	0.0
Ostomy takedown	6	25	4.5
Small bowel resection	11	23	5.6
Resection of retroperitoneal mass	8	17	4.6
Lung resection	9	38 (3.3)	5.7
Laparoscopic hiatal hernia repair	15 (3.9)	43 (3.7)	0.9
Aortobifemoral bypass	1	5	2.9
Endovascular aortic procedure	6	22	2.6
Open inguinal hernia repair	3	18	7.2
Laparoscopic cholecystectomy	10	30	0.0
Laparoscopic appendectomy	2	12	5.9
Lysis of adhesions	2	6	0.0
Hepaticojejunostomy	2	6	0.0

*ASA*, American Society of Anesthesiologists; *COPD*, chronic obstructive pulmonary disease; *MIS*, minimally invasive surgery; *SD*, standard deviation; *SPAR*, Surgical Prehabilitation and Readiness.
