# Supplementary material for: The benefits of a department-wide prehabilitation program: A propensity score match analysis
Source: Surgery. Author manuscript; Available in PMC 2026 Apr 19. (PMC13092355; doi:10.1016/j.surg.2025.109489)
Supplement: 1 [file NIHMS2162041-supplement-1.docx]

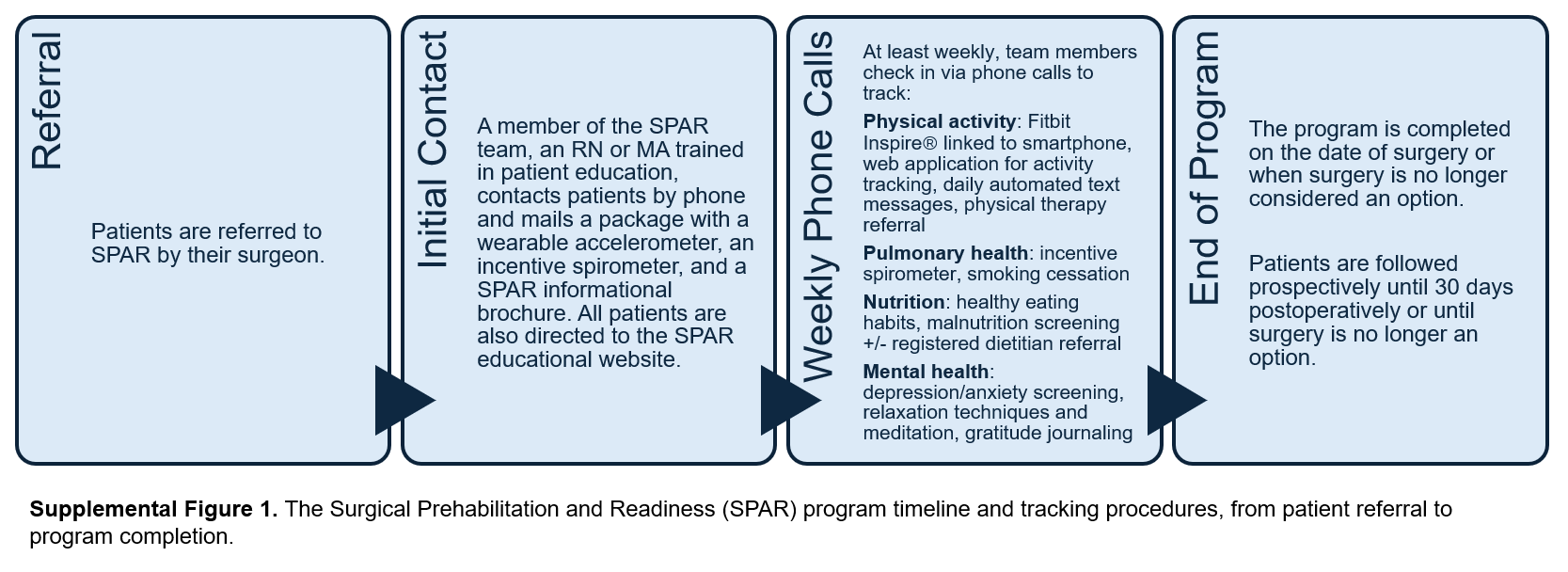


| **Supplemental Table 1.** CPT Codes Used for Surgical Procedures | |
| --- | --- |
| **Operative procedure** | **CPT code** |
| Abdominal aortic aneurysm repair, open^1^ | 35081, 35102, 35082, 35103, 35091, 35092 |
| Adrenalectomy, MIS^2^ | 60650 |
| Aortobifemoral bypass^3^ | 35646 |
| Bilateral salpingoophorectomy^4^ | 58954 |
| Colectomy, MIS^5^ | 44206, 44207 44208, 44210, 44204 |
| Colectomy, open^5^ | 44145, 44146, 44147, 44150 |
| Cystectomy^6^ | 51590, 51595, 51596 |
| Deep inferior epigastric perforator flap^7^ | 19364 |
| Distal gastrectomy^8^ | 43631, 43632, 43633, 43634 |
| Distal pancreatectomy and splenectomy^9^ | 48140, 48145, 48146 |
| Endovascular aortic procedure^1^ | 34800, 34802, 34803, 34804, 34805 |
| Esophagectomy^10^ | 43107, 43124, 43117, 43287, 43121, 43122, 43286, 43112, 43288 |
| Hepaticojejunostomy^11^ | 47760, 47765, 47780, 47785 |
| Incisional hernia repair with mesh^12^ | 49560, 49561, 49565, 49566, 49568, 49570, 49572, 49580, 49582, 49585, 49587, 49590 |
| Laparoscopic cholecystectomy^13^ | 47562, 47563, 47564, 47600, 47605, 47610 |
| Laparoscopic hiatal hernia repair^14^ | 43280, 43281, 43282 |
| Lung resection^15^ | 32440, 32442, 32445, 32480, 32484 |
| Major hepatectomy^16^ | 47125, 47130, 47122 |
| Mediastinal tumor resection^17^ | 39220 |
| Ostomy takedown^18^ | 44620, 44625, 44626 |
| Pancreaticoduodenectomy^19^ | 48150, 48152, 48153, 48154 |
| Partial hepatectomy^16^ | 47120 |
| Rectal resection, MIS^5^ | 44211, 44212, 45395, 45397 |
| Rectal resection, open^5^ | 45110, 44155, 44156, 45111, 45114, 45123, 45119, 45112, 45113 |
| Resection of retroperitoneal mass^20^ | 49203, 49204, 49205 |
| Small bowel resection^21^ | 44120, 44125, 44130 |
| Thyroidectomy^22^ | 60210, 60212, 60220, 60225, 60200, 60240, 60271, 60252, 60254, 60260 |
| Total gastrectomy^8^ | 43620, 43621, 43622 |
| Excision of infected wound^23^ | 35907 |
| Inguinal hernia repair, open^24^ | 49505, 49507, 49520, 49521, 49525 |
| Prostatectomy^25^ | 55866 |
| Lysis of adhesions^26^ | 44005, 44180, 50715, 58660 |
| Lymphadenectomy^27^ | 38760 |
| Scrotal Repair^28^ | 55180 |
| Appendectomy, laparoscopic^29^ | 44970 |
| Appendectomy, open^29^ | 44950 |
| Ureteral Repair^30^ | 52344, 52005, 52352, 52356 |
| Skin Excision^31^ | 15830 |
| Mastectomy^32^ | 19303, 19304, 19305, 19306, 19307 |
| Hyperthermic intraperitoneal chemotherapy (HIPEC)^33^ | 77605, 96445, 96446 |
| Heart valve replacement^34^ | 33405, 33406, 33407, 33408, 33409, 33410, 33411, 33412, 33430 |
| Wound debridement^35^ | 11044 |
| MIS, minimally invasive surgery | |


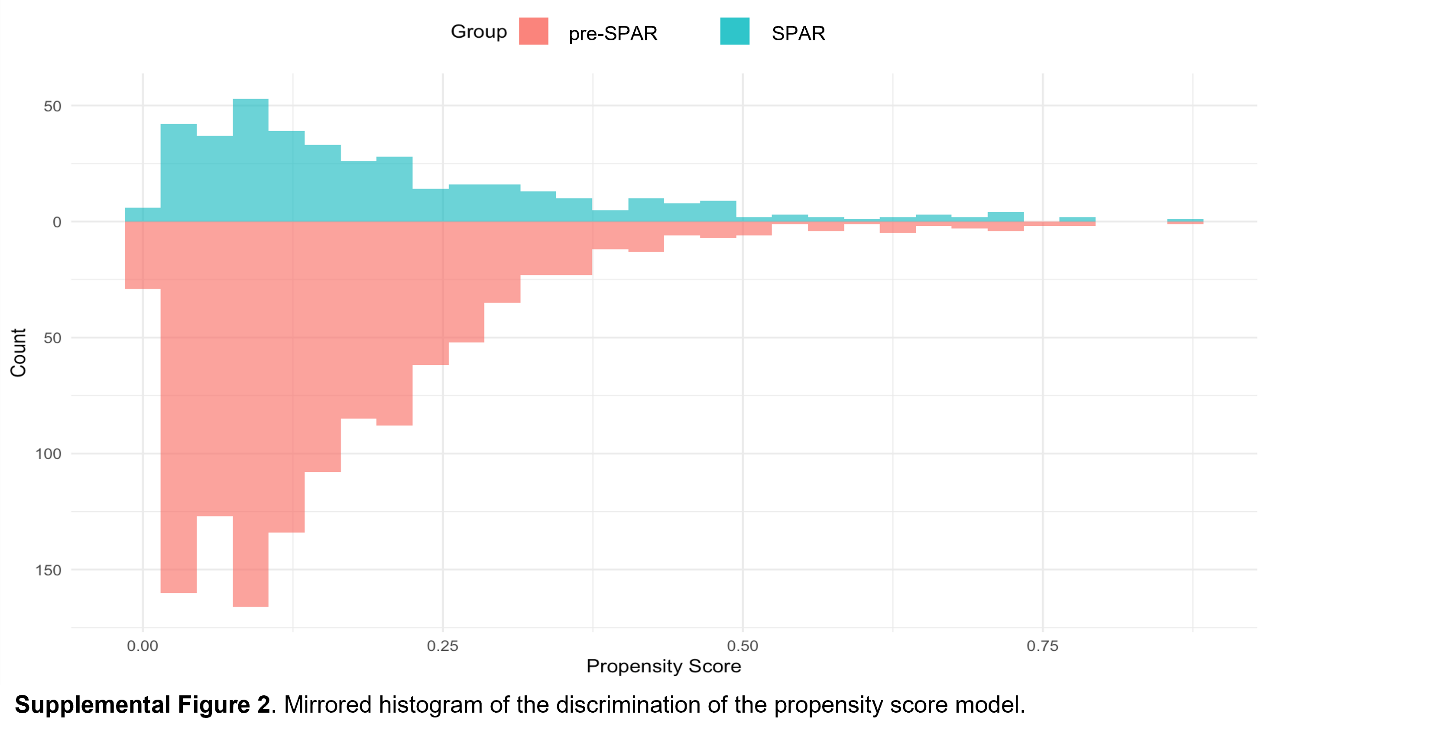


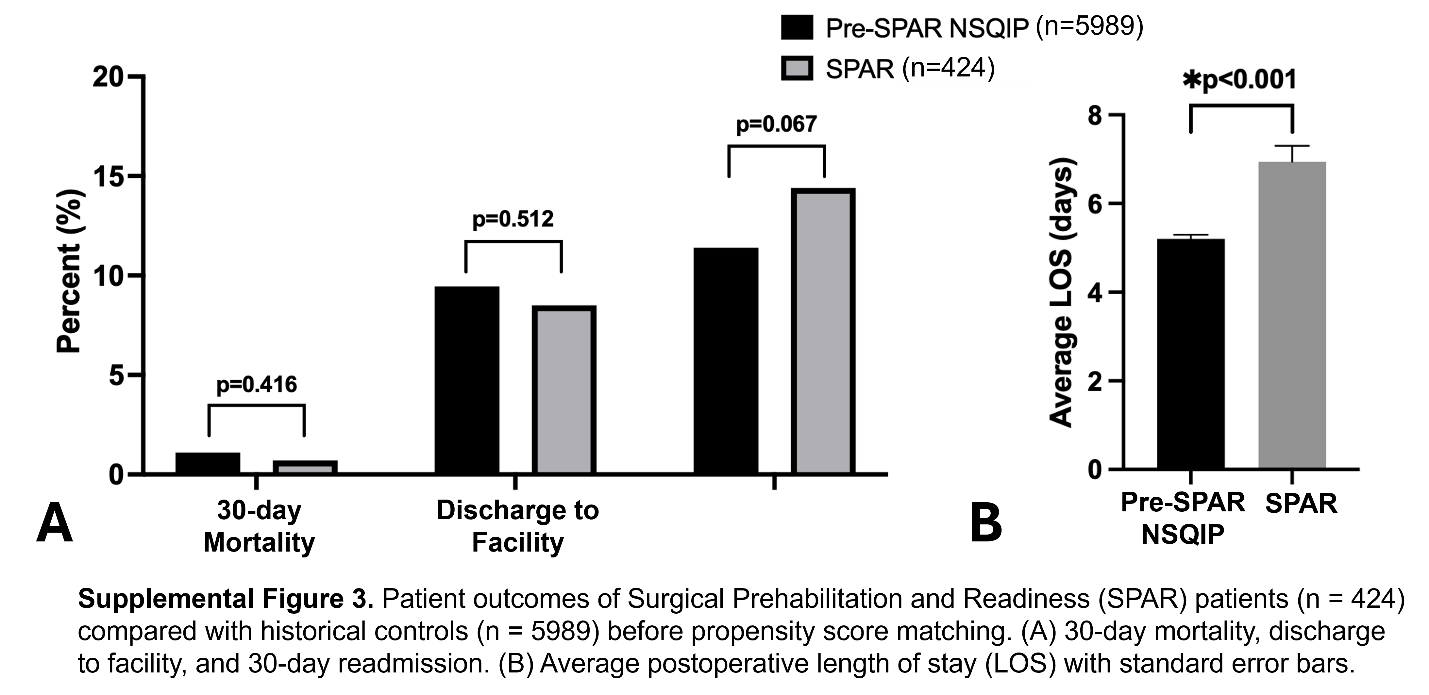


| **Supplemental Table 2.** Characteristics of SPAR Patients and Propensity Score–Matched Pre-SPAR NSQIP Patients > Median Age (69.9 years) | | | |
| --- | --- | --- | --- |
|  |  |  |  |
| **Characteristic** | **SPAR patients (n = 181)** | **Matched pre-SPAR NSQIP patients (n = 543)** | **Standardized Difference, %** |
| Age, y, mean (SD) | 76.6 (4.7) | 76.6 (5.2) | 0.7 |
| Sex, f, n, (%) | 81 (44.8) | 246 (45.3) | 1.1 |
| Non-White race, n (%) | 22 (12.2) | 58 (10.7) | 4.6 |
| Smoking, n (%) | 23 (12.7) | 69 (12.7) | 0.0 |
| ASA status ≥ 3, n (%) | 161 (89.0) | 476 (87.7) | 4.0 |
| Independent function, n (%) | 174 (96.1) | 528 (97.2) | 6.2 |
| Obesity, n (%) | 56 (30.9) | 167 (30.8) | 0.4 |
| Dyspnea, n (%) | 56 (30.9) | 150 (27.6) | 7.3 |
| Diabetes, n (%) | 45 (24.9) | 120 (22.1) | 6.5 |
| Steroid use, n (%) | 21 (11.6) | 48 (8.8) | 9.1 |
| Hypertension, n (%) | 124 (68.5) | 374 (68.9) | 0.8 |
| Congestive heart failure, n (%) | 12 (6.6) | 38 (7.0) | 1.5 |
| COPD, n (%) | 24 (13.3) | 60 (11.0) | 6.8 |
| Disseminated Cancer, n (%) | 4 (2.2) | 18 (3.3) | 6.7 |
| Operation type | | | |
| Pancreaticoduodenectomy, n (%) | 36 (19.9) | 102 (18.8) | 2.8 |
| Distal pancreatectomy and splenectomy, n (%) | 18 (9.9) | 42 (7.7) | 7.8 |
| MIS colectomy, n (%) | 24 (13.3) | 86 (15.8) | 7.3 |
| Open colectomy, n (%) | 6 (3.3) | 16 (2.9) | 2.1 |
| Open rectal resection, n (%) | 3 (1.7) | 8 (1.5) | 1.5 |
| MIS rectal resection, n (%) | 2 (1.1) | 10 (1.8) | 6.1 |
| Incisional hernia repair with mesh, n (%) | 6 (3.3) | 26 (4.8) | 7.4 |
| Cystectomy, n (%) | 25 (13.8) | 68 (12.5) | 3.8 |
| Esophagectomy, n (%) | 12 (6.6) | 43 (7.9) | 5.0 |
| Open abdominal aortic aneurysm repair, n (%) | 1 (0.6) | 7 (1.3) | 7.7 |
| Total gastrectomy, n (%) | 1 (0.6) | 2 (0.4) | 2.7 |
| Distal gastrectomy, n (%) | 5 (2.8) | 11 (2.0) | 4.8 |
| Partial hepatectomy, n (%) | 7 (3.9) | 16 (2.9) | 5.1 |
| Ostomy takedown, n (%) | 5 (2.8) | 11 (2.0) | 4.8 |
| Small bowel resection, n (%) | 2 (1.1) | 6 (1.1) | 0.0 |
| Resection of retroperitoneal mass, n (%) | 2 (1.1) | 8 (1.5) | 3.3 |
| Lung resection, n (%) | 5 (2.8) | 19 (3.5) | 4.2 |
| Laparoscopic hiatal hernia repair, n (%) | 9 (5.0) | 25 (4.6) | 1.7 |
| Endovascular aortic procedure, n (%) | 5 (2.8) | 19 (3.5) | 4.2 |
| Open inguinal hernia repair, n (%) | 2 (1.1) | 3 (0.6) | 6.1 |
| Laparoscopic cholecystectomy, n (%) | 2 (1.1) | 6 (1.1) | 0.0 |
| Laparoscopic appendectomy, n (%) | 2 (1.1) | 6 (1.1) | 0.0 |
| Hepatojejunostomy, n (%) | 1 (0.6) | 3 (0.6) | 0.0 |
| ASA, American Society of Anesthesiologists; MIS, minimally invasive surgery; SPAR, Surgical Prehabilitation and Readiness. | | | |

| **Supplemental Table 3.** Characteristics of SPAR Patients and Propensity Score–Matched Pre-SPAR NSQIP Patients ≤ Median Age (69.9 years) | | | | |
| --- | --- | --- | --- | --- |
|  |  |  |  |  |
| **Characteristic** | **SPAR patients (n = 198)** | **Matched pre-SPAR NSQIP patients (n = 594)** | **Standardized Difference, %** |  |
| Age, y, mean (SD) | 60.5 (9.6) | 59.7 (8.9) | 9.2 |  |
| Sex, f, n, (%) | 81 (40.9) | 261 (43.9) | 6.1 |  |
| Non-White race, n (%) | 21 (10.6) | 68 (11.4) | 2.7 |  |
| Smoking, n (%) | 63 (31.8) | 179 (30.1) | 3.6 |  |
| ASA status ≥ 3, n (%) | 159 (80.3) | 457 (76.9) | 8.2 |  |
| Independent function, n (%) | 192 (97.0) | 582 (98.0) | 6.4 |  |
| Obesity, n (%) | 90 (45.5) | 278 (46.8) | 2.7 |  |
| Dyspnea, n (%) | 53 (26.8) | 140 (23.6) | 7.4 |  |
| Diabetes, n (%) | 44 (22.2) | 152 (25.6) | 7.9 |  |
| Steroid use, n (%) | 33 (16.7) | 84 (14.1) | 7.0 |  |
| Hypertension, n (%) | 106 (53.5) | 316 (53.2) | 0.7 |  |
| Congestive heart failure, n (%) | 14 (7.1) | 40 (6.7) | 1.3 |  |
| COPD, n (%) | 28 (14.1) | 68 (11.4) | 8.1 |  |
| Disseminated Cancer, n (%) | 16 (8.1) | 49 (8.2) | 0.6 |  |
| Operation type |  |  |  |  |
| Pancreaticoduodenectomy, n (%) | 31 (15.7) | 101 (17.0) | 3.6 |  |
| Distal pancreatectomy and splenectomy, n (%) | 17 (8.6) | 48 (8.1) | 1.8 |  |
| MIS colectomy, n (%) | 18 (9.1) | 52 (8.8) | 1.2 |  |
| Open colectomy, n (%) | 7 (3.5) | 18 (3.0) | 2.8 |  |
| Open rectal resection, n (%) | 3 (1.5) | 12 (2.0) | 3.8 |  |
| MIS rectal resection, n (%) | 8 (4.0) | 20 (3.4) | 3.6 |  |
| Incisional hernia repair with mesh, n (%) | 6 (3.0) | 24 (4.0) | 5.5 |  |
| Cystectomy, n (%) | 26 (13.1) | 67 (11.3) | 5.7 |  |
| Esophagectomy, n (%) | 20 (10.1) | 59 (9.9) | 0.6 |  |
| Open abdominal aortic aneurysm repair, n (%) | 3 (1.5) | 9 (1.5) | 0.0 |  |
| Deep inferior epigastric perforator flap, n (%) | 3 (1.5) | 11 (1.9) | 2.6 |  |
| Total gastrectomy, n (%) | 3 (1.5) | 6 (1.0) | 4.5 |  |
| Distal gastrectomy, n (%) | 7 (3.5) | 12 (2.0) | 9.2 |  |
| Major hepatectomy, n (%) | 3 (1.5) | 12 (2.0) | 3.8 |  |
| Partial hepatectomy, n (%) | 8 (4.0) | 33 (5.6) | 7.1 |  |
| Ostomy takedown, n (%) | 1 (0.5) | 2 (0.3) | 2.6 |  |
| Small bowel resection, n (%) | 9 (4.5) | 19 (3.2) | 7.0 |  |
| Resection of retroperitoneal mass, n (%) | 4 (2.0) | 9 (1.5) | 3.8 |  |
| Lung resection, n (%) | 3 (1.5) | 15 (2.5) | 7.2 |  |
| Laparoscopic hiatal hernia repair, n (%) | 5 (2.5) | 18 (3.0) | 3.1 |  |
| Aortobifemoral bypass, n (%) | 1 (0.5) | 3 (0.5) | 0.0 |  |
| Endovascular aortic procedure, n (%) | 1 (0.5) | 5 (0.8) | 4.1 |  |
| Open inguinal hernia repair, n (%) | 1 (0.5) | 3 (0.5) | 0.0 |  |
| Laparoscopic cholecystectomy, n (%) | 7 (3.5) | 28 (4.7) | 5.9 |  |
| Lysis of adhesions, n (%) | 2 (1.0) | 6 (1.0) | 0.0 |  |
| Hepatojejunostomy, n (%) | 1 (0.5) | 2 (0.3) | 2.6 |  |
| ASA, American Society of Anesthesiologists; MIS, minimally invasive surgery; SPAR, Surgical Prehabilitation and Readiness. | | | | |

**Supplemental References**

1. Soden PA, Zettervall SL, Ultee KH, et al. Patient selection and perioperative outcomes are similar between targeted and nontargeted hospitals (in the National Surgical Quality Improvement Program) for abdominal aortic aneurysm repair. *J Vasc Surg.* 2017;65(2):362-371.

2. Elfenbein DM, Scarborough JE, Speicher PJ, Scheri RP. Comparison of laparoscopic versus open adrenalectomy: results from American College of Surgeons-National Surgery Quality Improvement Project. *J Surg Res.* 2013;184(1):216-220.

3. Madenci AL, Ozaki CK, Gupta N, et al. Perioperative outcomes of elective inflow revascularization for lower extremity claudication in the American College of Surgeons National Surgical Quality Improvement Program database. *Am J Surg.* 2016;212(3):461-467 e462.

4. Manning-Geist B, Cathcart AM, Sullivan MW, et al. Predictive validity of American College of Surgeons: National Surgical Quality Improvement Project risk calculator in patients with ovarian cancer undergoing interval debulking surgery. *Int J Gynecol Cancer.* 2021;31(10):1356-1362.

5. Sharp SP, Malizia R, Skancke M, et al. A NSQIP analysis of trends in surgical outcomes for rectal cancer: What can we improve upon? *Am J Surg.* 2020;220(2):401-407.

6. Reese SW, Ji E, Paciotti M, et al. Risk factors and reasons for reoperation after radical cystectomy. *Urol Oncol.* 2020;38(4):269-277.

7. Massenburg BB, Sanati-Mehrizy P, Ingargiola MJ, et al. Flap Failure and Wound Complications in Autologous Breast Reconstruction: A National Perspective. *Aesthetic Plast Surg.* 2015;39(6):902-909.

8. Jeong Y, Mahar AL, Coburn NG, et al. Outcomes of Non-curative Gastrectomy for Gastric Cancer: An Analysis of the American College of Surgeons National Surgical Quality Improvement Program (ACS-NSQIP). *Ann Surg Oncol.* 2018;25(13):3943-3949.

9. Kelly KJ, Greenblatt DY, Wan Y, et al. Risk stratification for distal pancreatectomy utilizing ACS-NSQIP: preoperative factors predict morbidity and mortality. *J Gastrointest Surg.* 2011;15(2):250-259, discussion 259-261.

10. Zheng R, Tham EJH, Rios-Diaz AJ, et al. A 10-year ACS-NSQIP Analysis of Trends in Esophagectomy Practices. *J Surg Res.* 2020;256:103-111.

11. Ismael HN, Cox S, Cooper A, Narula N, Aloia T. The morbidity and mortality of hepaticojejunostomies for complex bile duct injuries: a multi-institutional analysis of risk factors and outcomes using NSQIP. *HPB (Oxford).* 2017;19(4):352-358.

12. Savitch SL, Shah PC. Closing the gap between the laparoscopic and open approaches to abdominal wall hernia repair: a trend and outcomes analysis of the ACS-NSQIP database. *Surg Endosc.* 2016;30(8):3267-3278.

13. Fagenson AM, Powers BD, Zorbas KA, et al. Frailty Predicts Morbidity and Mortality After Laparoscopic Cholecystectomy for Acute Cholecystitis: An ACS-NSQIP Cohort Analysis. *J Gastrointest Surg.* 2021;25(4):932-940.

14. Mungo B, Molena D, Stem M, et al. Thirty-day outcomes of paraesophageal hernia repair using the NSQIP database: should laparoscopy be the standard of care? *J Am Coll Surg.* 2014;219(2):229-236.

15. Chudgar N, Yan S, Hsu M, et al. The American College of Surgeons Surgical Risk Calculator performs well for pulmonary resection: A validation study. *J Thorac Cardiovasc Surg.* 2022;163(4):1509-1516 e1501.

16. Leigh N, Williams GA, Strasberg SM, et al. Increased Morbidity and Mortality After Hepatectomy for Colorectal Liver Metastases in Frail Patients is Largely Driven by Worse Outcomes After Minor Hepatectomy: It's Not "Just a Wedge". *Ann Surg Oncol.* 2022;29(9):5476-5485.

17. Fadayomi AB, Iniguez CEB, Chowdhury R, et al. Propensity Score Adjusted Comparison of Minimally Invasive versus Open Thymectomy in the Management of Early Stage Thymoma. *Thorac Cardiovasc Surg.* 2018;66(4):352-358.

18. De Paula TR, Nemeth S, Kiran RP, Keller DS. Predictors of complications from stoma closure in elective colorectal surgery: an assessment from the American College of Surgeons National Surgical Quality Improvement Program (ACSNSQIP). *Tech Coloproctol.* 2020;24(11):1169-1177.

19. Panni RZ, Guerra J, Hawkins WG, et al. National Pancreatic Fistula Rates after Minimally Invasive Pancreaticoduodenectomy: A NSQIP Analysis. *J Am Coll Surg.* 2019;229(2):192-199 e191.

20. Judge SJ, Lata-Arias K, Yanagisawa M, et al. Morbidity, mortality and temporal trends in the surgical management of retroperitoneal sarcoma: An ACS-NSQIP follow up analysis. *J Surg Oncol.* 2019;120(4):753-760.

21. Horsey ML, Lai D, Herur-Raman A, et al. Open versus minimally invasive small bowel resection for Crohn's disease: a NSQIP retrospective review and analysis. *Surg Endosc.* 2022;36(8):6278-6284.

22. Sahli ZT, Ansari G, Gurakar M, et al. Thyroidectomy in older adults: an American College of Surgeons National Surgical Quality Improvement Program study of outcomes. *J Surg Res.* 2018;229:20-27.

23. Chaudhry SA, Rosenfeld ES, Glousman BN, Sparks AD, Lala S, Macsata R, Amdur R, Sidawy AN, Nguyen BN. Dependent functional status rather than age is a better predictor of adverse outcomes after excision of an infected abdominal aortic graft. J Vasc Surg. 2022 Apr;75(4):1413-1421.

24. Madion M, Goldblatt MI, Gould JC, Higgins RM. Ten-year trends in minimally invasive hernia repair: a NSQIP database review. Surg Endosc. 2021 Dec;35(12):7200-7208.

25. Harris AM, James A, Dugan A, Bylund J. Increased Operative Duration of Minimally Invasive Prostatectomy is Associated with Significantly Increased Risk of 30-Day Morbidity. Urol Pract. 2020 Jan;7(1):21-27.

26. Mavros MN, Bohnen JD, Ramly EP, Velmahos GC, Yeh DD, de Moya M, Fagenholz P, King DR, Lee J, Kaafarani HM. Intraoperative Adverse Events: Risk Adjustment for Procedure Complexity and Presence of Adhesions Is Crucial. J Am Coll Surg. 2015 Aug;221(2):345-53.

27. Chua KJ, Balraj V, Patel HV, Srivastava A, Doppalapudi SK, Elsamra SE, Jang TL, Singer EA, Ghodoussipour SB. Wound Complication Rates after Inguinal Lymph Node Dissection: Contemporary Analysis of the NSQIP Database. J Am Coll Surg. 2023 Jan 1;236(1):18-25.

28. Saxena N, Jolly D, Wu CA, Boskey ER, Ganor O. Comparing scrotoplasty complication rates in transgender and cisgender men: An ACS NSQIP study. JPRAS Open. 2023 Apr 3;36:55-61.

29. Page AJ, Pollock JD, Perez S, Davis SS, Lin E, Sweeney JF. Laparoscopic versus open appendectomy: an analysis of outcomes in 17,199 patients using ACS/NSQIP. J Gastrointest Surg. 2010 Dec;14(12):1955-62.

30. Davidson J, Ding Y, Chan E, Dave S, Bjazevic J, Filler G, Wang PZT. Postoperative

outcomes of ureteroscopy for pediatric urolithiasis: A secondary analysis of the National

Surgical Quality Improvement Program Pediatric. J Pediatr Urol. 2021 Oct;17(5):649.e1-

649.e8.

31. Kuruoglu D, Salinas CA, Tran NV, Nguyen MT, Martinez-Jorge J, Bite U, Harless CA,

Sharaf B. Abdominal Panniculectomy: An Analysis of Outcomes in 238 Consecutive

Patients over 10 Years. Plast Reconstr Surg Glob Open. 2021 Nov 24;9(11):e3955.

32. Hu VJ, McCleary SP, Smullin CP, Rosales Morales R, Da Lio AL. Current Trends in Breast

Reconstruction following Bilateral Prophylactic Mastectomy. Plast Reconstr Surg Glob

Open. 2022 Apr 18;10(4):e4277.

33. Jafari MD, Halabi WJ, Stamos MJ, Nguyen VQ, Carmichael JC, Mills SD, Pigazzi A.

Surgical outcomes of hyperthermic intraperitoneal chemotherapy: analysis of the

American college of surgeons national surgical quality improvement program. JAMA

Surg. 2014 Feb;149(2):170-5.

34. Dyas AR, Bronsert MR, Henderson WG, Stuart CM, Pradhan N, Colborn KL, Cleveland JC

Jr, Meguid RA. A comparison of the National Surgical Quality Improvement Program

and the Society of Thoracic Surgery Cardiac Surgery preoperative risk models: a cohort

study. Int J Surg. 2023 Aug 1;109(8):2334-2343.

35. Hornor MA, Ma M, Zhou L, Cohen ME, Rosenthal RA, Russell MM, Ko CY. Enhancing the

American College of Surgeons NSQIP Surgical Risk Calculator to Predict Geriatric

Outcomes. J Am Coll Surg. 2020 Jan;230(1):88-100.
